# Transcatheter Mitral Valve-in-Valve Implantations Using Inverted J-Valve

**DOI:** 10.3389/fcvm.2022.896639

**Published:** 2022-06-23

**Authors:** Lulu Liu, Bowen Xiao, Binggang Wu, Yingqiang Guo

**Affiliations:** Department of Cardiovascular Surgery, West China Hospital, Sichuan University, Chengdu, China

**Keywords:** transcatheter mitral valve implantation, valve-in-valve, J-valve, structural valve deterioration, transapical

## Abstract

**Background:**

As bioprosthetic valves are being widely used, the incidence of structural valve deterioration increases, as well as the need for reoperation. Transcatheter mitral valve-in-valve implantations are being increasingly adopted as an alternative to redo-surgical mitral replacement for patients with high surgical risks. This study reports a series of transcatheter mitral valve-in-valve implantations using inverted J-valves.

**Methods:**

From April 2019 to September 2021, 17 symptomatic high-risk patients with mitral bioprosthetic valve dysfunction underwent transapical transcatheter mitral valve-in-valve implantations using inverted J-valves at our institution.

**Results:**

The median age was 70 years, with 76.5% being female. The median Society of Thoracic Surgeons predicted risk of mortality (STS PROM) was 17.2% (8.7–82.24%). All patients had successful transapical transcatheter mitral valve-in-valve implantations except for one intraoperative death due to left ventricle rupture. Four patients underwent simultaneous transcatheter aortic valve implantation, two of which had valve-in-valve transcatheter aortic valve implantation. There was no major complication except one case of bleeding. Thirty-day mortality was 11.8% (2/17), and 90-days mortality was 23.5% (4/17). Percentages of patients with New York Heart Association class III/IV symptoms decreased from 100 (17/17) to 20% (3/15) at 30-days. Median mitral inflow velocity was 1.95 mm/s at 30 days, compared to 2.7 mm/s at baseline. Median mitral valve effective orifice area increases from 1.5 mm at baseline to 1.85 mm at 30 days.

**Conclusion:**

Transcatheter transapical valve-in-valve implantations with J-valve can be a plausible solution to failed mitral bioprosthesis with acceptable results for high-risk patients.

## Introduction

As bioprosthetic valves are being increasingly adopted, structural valve deterioration becomes a challenge for long-term prognosis. The introduction of valve-in-valve TAVI marks the beginning of a new era for failed bioprosthetic valves (1–4). Three-year follow-up results from PARTNER 2 registry (5) demonstrate favorable survival, sustained improved hemodynamic status, and excellent functional and quality-of-life outcomes using valve-in-valve TAVR for patients with structural valve deterioration.

However, the use of valve-in-valve transcatheter mitral valve replacement (TMVIV) remains controversial compared to repeat surgical interventions (6, 7), especially in patients with small-sized failed surgical bioprostheses. Current guidelines (8, 9) acknowledge TMVIV as an alternative to surgical re-implantation in Comprehensive Valve Centers for patients with high surgical risks. Kamioka et al. (10) find similar clinical and echocardiographic outcomes after surgical redo mitral valve replacement and transcatheter mitral valve-in-valve therapy.

Usage of inverted TAVR prosthesis in TMVIV has been widely reported (11, 12). Mid-term reports from multiple cohorts have shown acceptable results using SAPIEN 3 [(Edwards Lifesciences), Melody (Medtronic, Minneapolis, MN), Lotus (Boston Scientific, Natick, MA, USA) and Direct Flow (Direct Flow Medical Inc., Santa Rosa, CA, USA)] (13–17). Most procedures are performed *via* a transapical or transseptal approach. The transapical route provides coaxial alignment and therefore reduces the risk of malposition and migration, along with left ventricular outflow tract obstruction. In addition, the transapical access offers an intergrated solution for patients in need of additional aortic valve intervention.

J-valve (Jie Cheng Medical Technologies, Suzhou, China) is a second-generation self-expanding bioprosthetic valve designed for transapical TAVR. It has been approved by the China National Medical Products Administration for both aortic valve stenosis and regurgitation after proved effective and safe in the multicentered study. Lu et al. (18) and Wei et al. (19) reported their experience with TMVIV using J-valve in 26 and 21 patients, respectively. In this study, we report 17 cases of TMVIV using inverted J-valves.

## Methods

### Ethics Statement

The study protocol was approved by the West China Hospital Ethics Committees and Institutional Review Board, Sichuan, China. Written informed consent was obtained from all patients.

### Patients

Our retrospective cohort included 17 consecutive patients with mitral bioprosthetic valve dysfunction (regurgitation and/or stenosis) who underwent transapical transcatheter mitral valve-in-valve implantations using inverted J-valves at our institution between April 2019 and September 2021. Indications for redo mitral valve replacement were based on the 2014 American College of Cardiology/American Heart Association Guideline for the Management of Patients with Valvular Heart Disease (20). Patients were deemed unsuitable for re-operative mitral valve surgery because of excessive surgical risk after heart team discussion. Inclusion criteria for the procedure were the following: presence of a dysfunctional bioprosthesis in mitral position; STS score >8% or logistic EuroSCORE >10; Exclusion criteria for the procedure included left ventricular thrombus; cardiac tumors; presence of periprosthetic leak; prosthesis label size <25 or >31; active endocarditis; myocardial infarction or stroke within 1 month; severe coronary artery disease that requires revascularization; presence of contraindications for anticoagulation. Notably, patients with left atrial thrombosis were not excluded from the cohort, as the transapical device would have little impact on the thrombosis comparing to transeptal devices.

### Preprocedural Planning

All patients underwent clinical examinations, laboratory tests, echocardiography, and cardiac computed tomography before the procedure. The sizing of the J-valve was based on the label size of the previous bioprosthesis and the ring measurement on cardiac CT. Data on baseline characteristics, procedural details, and outcomes were retrospectively collected from the hospital information system. Transthoracic echocardiographic analysis was performed preoperatively, postoperatively, after implantation at 1 week and 1 month. Clinical follow-up was performed by the heart team at 1 month and 3months.

### Device and Procedure

**Figure 1:**
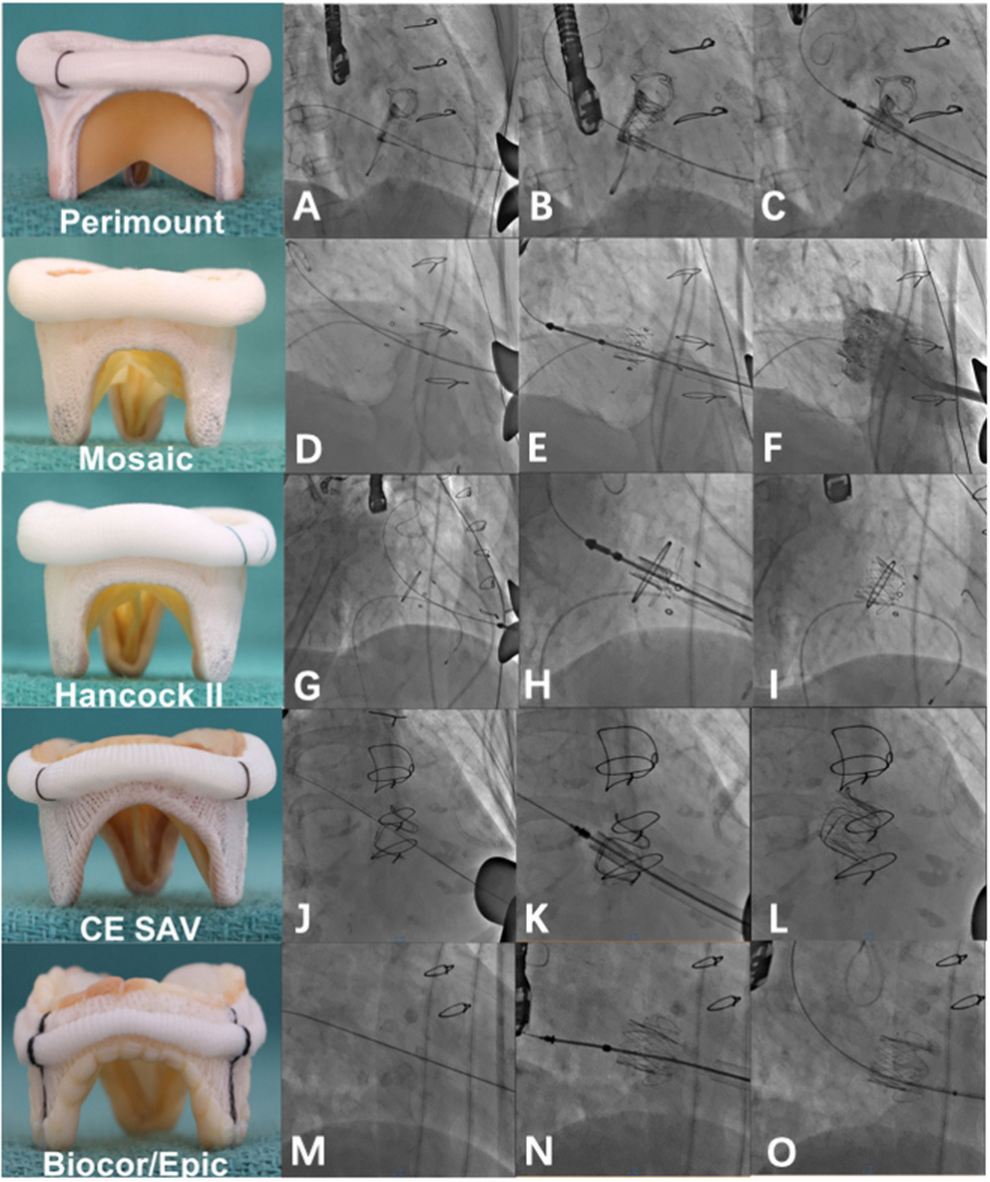
Valve-in-valve implantation of a J-valve into different degenerated bioprosthetic valves. **(A)** A degenerated EDW Perimount mitral valve prosthesis under fluoroscopy. **(B)** J-valve deployment into a degenerated EDW Perimount mitral valve prosthesis. **(C)** Post-implantation. **(D–F)** Valve-in-valve implantation of a J-valve into a degenerated Mosaic prosthesis. **(G–I)** Valve-in-valve implantation of a J-valve into a degenerated Hancock II prosthesis. **(J–L)** Valve-in-valve implantation of a J-valve into a degenerated CE SAV prosthesis. **(M–O)** Valve-in-valve implantation of a J-valve into a degenerated Epic prosthesis. Note that there is no radiolucent marker on the Epic prosthesis.

J-Valve^TM^ prosthetic valve (Figure 1) is originally a self-expanding TAVR device approved for both aortic stenosis and aortic regurgitation. Features of the J-Valve^TM^ system include a trifoliate porcine aortic valve, a self-expanding nitinol stent, three U-shaped anatomically oriented “graspers” for optimal positioning, and a polyester skirt covering the outer surface of the valve stent to minimize the risk of paravalvular leakage (21). The available sizes of the J-Valve were as follows: 21, 23, 25, 27, and 29 mm.

Transapical implantation of the J-Valve prosthesis was performed under general anesthesia by an interdisciplinary heart team in a hybrid operating room. The technique of the TMVIV procedure was similar to that of Lu et al. (18) and Wei et al. (19). All patients were kept on warfarin therapy with an INR goal of 2–3 for 3–6 months. Patients with atrial fibrillation received long-term warfarin for anticoagulation.

### Definitions

We used standardized endpoint criteria according to the Mitral Valve Academic Research Consortium (MVARC) for the data collection (22).

### Statistical Analysis

Statistical analysis was performed using the SPSS 20 (IBM, Armonk, NY, USA). After the normality test, continuous variables with normal distribution were described as mean (± standard deviation of the mean), and continuous variables without normal distribution were described as median (interquartile range, IQR). Categorical data were described as numbers (percentage). A value of *P* < 0.05 was considered statistically significant.

## Results

### Baseline Characteristics

From April 2019 to September 2021, 17 patients underwent transapical TMVIV procedures in our institution (Table 1). The median age of the patients was 70 years (IQR 9), with 76.5% (13/17 being female. All patients were symptomatic with New York Heart Association (NYHA) class III (17.6%)/IV (82.4%) heart failure. All patients were deemed unsuitable for conventional redo mitral valve replacement surgery by our heart team due to extreme surgical risk, with a median Society of Thoracic Surgeons predicted risk of mortality (STS PROM) of 17.2% (IQR 27.4, range 8.7–82.2%.) and median Euroscore II of 24.7 (IQR 31.6). Our patient cohort even included some critical patients. Seven patients (41.2%) were hospitalized in the Intensive Care Unit (ICU) before the procedure, and 4 of those (23.6%) were intubated. One patient had a cardiac arrest 2 days before the procedure, and she was on Extracorporeal Membrane Oxygenation (ECMO) and Continuous Renal Replacement Therapy (CRRT) support. Compassionate emergency surgery was performed on these 7 critical patients in ICU, while the other 10 patients received elective surgery.

**Table 1:** Baseline characteristics and procedural outcomes.

		***N* = 17**
Demographics	Age (years)	70 (9)
	Female (%)	13 (76.5%)
Medical history	Hypertension	4 (23.4%)
	Diabetes mellitus	10 (58.8%)
	Coronary artery disease	4 (23.5%)
	Pulmonary hypertension	13 (76.5%)
	History of heart failure	11 (64.7%)
	Atrial fibrillation	14 (82.4%)
	Pre-operation intubation	4 (23.5%)
	ICU hospitalization	7 (41.2%)
	Tricuspid regurgitation moderate or higher	12 (70.6%)
NYHA class	III	3 (17.6%)
	IV	14 (82.4%)
Risk score	Euroscore II	24.7 (31.6)
	STS	17.2 (27.4)
Mechanism of mitral valve failure	Regurgitation	11 (64.7%)
	Stenosis	4 (23.5%)
	Combined	2 (11.8%)
Previous procedure	Previous MVR (%)	10 (58.8%)
	Previous DVR (%)	7 (41.2%)
	Time from previous procedure (years)	10 (5)
	Previous mitral bioprosthesis size (mm)	25 (2)
Previous Device type	Hancock II	6 (35.2%)
	Epic	4 (23.6%)
	CE SAV	1 (5.9%)
	Mosaic	4 (23.6%)
	EDW Perimount	2 (11.8%)
Procedural details	Transapical access	17 (100%)
	TMVIV	13 (76.5%)
	TMVIV+TAVR	2 (11.8%)
	TMVIV+TAVIV	2 (11.8%)
	Replacing J-valve size (mm)	23 (2)
	Balloon pre-dilatation	4 (23.5%)
	Balloon post-dilatation	14 (82.4%)
	Device success	17 (100%)
	Procedural Success	16 (94.1%)
	Total procedure time (min)	82 (27)
	Fluoroscopy time	10.9 (6.4)
	Contrast dose (ml)	0 (34)
Procedural outcomes	Device success	17 (100%)
	Procedural Success	16 (94.1%)
	Prolonged ventilation, >24 h	6 (35.3%)
	Reintubation	2 (11.8%)
	Tracheotomy	3 (17.6%)
	Conversion to conventional surgery	0 (0%)
	LVOT obstruction	0 (0%)
	Valve embolization	0 (0%)
	Need for second valve implantation	0 (0%)
	Left ventricular perforation	1 (5.9%)
	Re-intervention	0 (0%)
30-day outcomes (*n* = 15)	Mitral inflow velocity (mm/s)	2.7 (0.5)
	Mitral valve EOA (cm^2^)	1.5 (1.2)
	Bleeding complication	1 (5.9%)
	NYHA class ≥ III	3 (20%)
	Stroke	0 (0%)
	New complete heart block	0 (0%)
	Procedure-related death	1 (5.9%)

*ICU, intensive care unit; NYHA, New York Heart Association; STS, Society of Thoracic Surgeons; MVR, mitral valve replacement; DVR, aortic and mitral valve replacement; TAVR, transcatheter aortic valve implantation; TAVIV, transcatheter aortic valve-in-valve implantation; TMVIV, transcatheter mitral valve-in-valve implantation; LVOT, left ventricle outflow tract; EOA, effective orifice area*.

A variety of mitral bioprosthesis failed in our patient cohort 5–15 years after implantation, with a median time from the previous procedure of 10 years (IQR 5). The mechanism of bioprosthetic valvular dysfunction was secondary to severe mitral regurgitation in 64.7% (*n* = 11) and stenosis in 23.5% (*n* = 4) patients. Two patients had combined regurgitation and stenosis. The median left ventricular ejection fraction (LVEF) was 70% (IQR 7). Median mitral inflow velocity was 2.7 mm/s (IQR 0.5), and median mitral valve EOA was 1.5 cm^2^ (IQR 1.2). Twelve patients (71.6%) had moderate or severe tricuspid regurgitation. Seven patients (41.2%) had previous DVR (double valve replacement, i.e., aortic and mitral valve replacement). Six patients (35.2%) implanted Hancock II bioprosthesis, 4 patients (23.6%) implanted Epic, 4 patients (23.6%) implanted Mosaic, 2 patients (11.8%) implanted Edwards Perimount, and 1 patient (5.9%) implanted CE SAV. The median size of the previous mitral bioprosthesis was 25 mm (IQR 2).

### Procedural Outcomes

All 17 patients had transapical TMVIV using J-valve, among which 2 patients (11.8%) had combined TAVR and 2 patients (11.8%) had combined valve-in-valve TAVR using J-valve. The median size of the J-valve was 23 mm (IQR 2). Balloon pre-dilatation was performed in 4 patients (23.5%), and balloon post-dilatation was performed in 14 patients (82.4%). One patient died immediately after balloon post-dilation due to left ventricular perforation. We presumed that the long stent of the Hancock II prosthesis was pushed to the left ventricle posterior wall during balloon post-dilation, leading to ventricular rupture. However, this presumption was not confirmed because the family refused an autopsy. Procedural success was achieved in the other 16 patients (94.1%). The median procedural time was 82 min (IQR 27). All patients are free from stroke, new complete heart block, LVOT obstruction, or valve embolization after the procedure. One patient had a bleeding complication. Six patients (35.3%) had prolonged ventilation over 24 h.

The first patient in our cohort was admitted to ICU before the procedure, and he had poor ventilation requiring intubation. After the procedure, the patient had reintubation with a tracheotomy. The patient had a prolonged intensive care unit stay of 16 days and died on day 25 due to in-hospital pneumonia. There was no other in-hospital mortality except for one intraoperative mortality described above. No patient was lost to follow-up at 90 days. Overall, 30-day mortality was 11.8% (2/17). One patient died 75 days after the procedure due to sudden cardiac death. Another patient died 90 days post-procedure due to cerebral hemorrhage. Overall, 90 days mortality was 23.5% (4/17). There was no other mortality at the last follow-up. No reintervention, conversion to conventional surgery, second valve implantation, or IABP was required.

### Hemodynamic Performance

Median mitral inflow velocity decreases from 2.7 mm/s (IQR 0.5) at baseline to 1.8 mm/s (IQR 0.5) 1-week post-procedure. At 30-days follow-up, the median mitral inflow velocity was 1.95 mm/s (IQR 0.5). Median mitral valve EOA increases to 2.1 cm^2^ (IQR 0.6) 1 week post-procedure, compared to a baseline level of 1.5 cm^2^ (IQR 1.2). The percentage of patients with NYHA functional class III and IV decreased from 100% before the procedure to 31.3% at 1 week and 20% 1 month after the procedure.

## Discussion

In this study, we report 17 cases of TMVIV using J-valve at West China Hospital, Chengdu, China. Our cohort included patients with higher risks (mean STS-PROM of 28.58 ± 19.96%) compared to the study from Lu et al. (18) (12.3 ± 8.3%) and Wei et al. (19) (12.03 ± 10.5%). More patients underwent concomitant TAVR or TAVIV (23.5%) in our cohort. The 30-day mortality (11.8%) was higher than what was reported in the above cohorts (0–3.8%), but it was still acceptable considering that 41.2% of our patients were hospitalized in ICU, and 23.5% were intubated, and one was on ECMO before the procedure. Our patients had a high burden of comorbidities at baseline (58.8% with diabetes mellitus, 64.7% with a history of heart failure, 82.4% with atrial fibrillation, 76.5% with pulmonary hypertension, and 52.9% with renal insufficiency), and yet the median post-operative hospitalization days (8 days) were comparable to the results in the above studies.

Mitral inflow velocity (mm/s) decreased from 2.7 (0.5) to 1.95 (0.5) mm/s at 30-days, and the mitral valve EOA (cm^2^) increased from 1.5 (1.2) to 1.85 (0.6) cm^2^. Improvement in clinical symptoms has been shown in our cohort, as the percentage of patients with NYHA functional class III and IV decreased from 100% before the procedure to 31.3% at 1 week and 20% at 1 month after the procedure, indicating that the left ventricular ejection may have appeared to be better than it was before MR correction, which is consistent with the report of previous studies (23–25). A significant and immediate reduction in pulmonary artery pressure following TMVIV implantation was observed 1 week following implantation, and the effect was continuing at a 1-month follow-up. Considering the high incidence of chronic lung disease (94.1%) in our cohort, correction of MR plays an important role in relieving pulmonary hypertension. We also observed a decrease in the percentage of patients with moderated or severe tricuspid regurgitation, from 12 (70.6%) to 5(33%) at 30 days, which may be the consequence of reduced pulmonary arterial pressure. Medvedofsky et al. (26) reported tricuspid regurgitation regression in patients with pulmonary hypertension in association with a remarkable right ventricular reverse remodeling. Sadeghi et al. (27) reported TR regression in patients undergoing successful pulmonary endarterectomy, frequently occurring despite persistent TA dilation and no change in valve coaptation. Our finding was consistent with their conclusion that functional tricuspid regurgitation may be reversed after pulmonary arterial pressure reduction. Therefore, indications for concomitant tricuspid valve intervention should be reconsidered in our cohort with 76.5% (13/17) of the patient being pulmonary hypertensive.

Importantly, significant paravalvular regurgitation was not observed following valve-in-valve implantation into mitral surgical bioprosthesis. 88.2 and 76.5% of our high-risk elderly patients were alive and well at 30-days and 90-days, respectively. One patient died intraoperatively due to left ventricular rupture during post-dilation. One patient was frail at baseline, and he died 25 days post-procedure due to a pulmonary infection. Another two patients died of sudden cardiac death and cerebrovascular hemorrhage at 75 and 90 days, respectively. Our experience with these mortality cases highlights the importance of careful decision-making when selecting very high-risk patients, and that balloon valvuloplasty should be adopted with discretion. In addition, we presumed that the long stent of the Hancock II prosthesis was pushed to the left ventricle posterior wall during balloon post-dilation, leading to ventricular rupture. This reminds cardiac surgeons not to implant the surgical bioprosthesis stent in proximity to the left ventricle posterior wall. The adverse event rate was low, and most patients have discharged within 14 days post-procedure. No structural failure of transcatheter valves or valve reoperation was observed in our relatively short follow-up. However, studies in a larger cohort with a longer follow-up are needed in the future.

We adopted transapical access in all procedures, which allows a short, direct, and coaxial route for TMVIV. Nevertheless, studies from Yoon et al. (17) showed that the procedural and clinical outcomes of the transseptal approach were comparable to those of the transapical approach, except for the more frequent requirement of closure of the iatrogenic atrial septal defect. The transapical route also allows an integrated solution to concomitant TAVR or valve-in-valve TAVR, which was performed in 4 (23.5%) patients. Also, the price and reimbursement policies make J-valve a more affordable choice compared to Sapien 3 in China. J-valve was the only commercially available device for transcatheter mitral valve replacement in China until Sapien 3 (Edwards Lifesciences) was approved by China National Medical Products Administration in June 2021. Besides a lack of experience in transseptal TMVR, the presence of a thickened fibrotic septum due to previous surgical intervention was another reason why the author favored the transapical route over the transeptal approach after reviewing the surgical records, which documented septum incision and sutures in most cases. In addition, a few patients in our cohort had left atrial thrombus identified before or during the procedure, which mandates transapical access.

## Conclusion

Transapical TMVIV is a feasible and reproducible procedure. Our early experience with this strategy using J-valve is encouraging.

## Data Availability Statement

The raw data supporting the conclusions of this article will be made available by the authors, without undue reservation.

## Ethics Statement

The studies involving human participants were reviewed and approved by West China Hospital Ethics Committees and Institutional Review Board, Sichuan, China. The patients/participants provided their written informed consent to participate in this study.

## Author Contributions

LL and BX: conception and design, data analysis, and interpretation. YG: administrative support. LL, BX, and BW: collection and assembly of data. All authors wrote the manuscript and approved the final manuscript.

## Funding

This work was funded by the 1.3.5 project for disciplines of excellence, West China Hospital, Sichuan University.

## Conflict of Interest

The authors declare that the research was conducted in the absence of any commercial or financial relationships that could be construed as a potential conflict of interest.

## Publisher's Note

All claims expressed in this article are solely those of the authors and do not necessarily represent those of their affiliated organizations, or those of the publisher, the editors and the reviewers. Any product that may be evaluated in this article, or claim that may be made by its manufacturer, is not guaranteed or endorsed by the publisher.

## References

[1] Deharo P Bisson A Herbert J Lacour T Etienne CS Porto A. Transcatheter valve-in-valve aortic valve replacement as an alternative to surgical re-replacement. J Am Coll Cardiol. (2020) 76:489–99. 10.1016/j.jacc.2020.06.010 32731926

[2] Dvir D Webb J Brecker S Bleiziffer S Hildick-Smith D Colombo A. Transcatheter aortic valve replacement for degenerative bioprosthetic surgical valves: results from the global valve-in-valve registry. Circulation. (2012) 126:2335–44. 10.1161/CIRCULATIONAHA.112.104505 23052028

[3] Dvir D Webb JG Bleiziffer S Pasic M Waksman R Kodali S. Transcatheter aortic valve implantation in failed bioprosthetic surgical valves. JAMA. (2014) 312:162–70. 10.1001/jama.2014.7246 25005653

[4] Deeb GM Chetcuti SJ Reardon MJ Patel HJ Grossman PM Schreiber T. 1-Year results in patients undergoing transcatheter aortic valve replacement with failed surgical bioprostheses. JACC Cardiovasc Interv. (2017) 10:1034–44. 10.1016/j.jcin.2017.03.018 28521921

[5] Webb JG Murdoch DJ Alu MC Cheung A Crowley A Dvir D. 3-Year outcomes after valve-in-valve transcatheter aortic valve replacement for degenerated bioprostheses: the PARTNER 2 registry. J Am Coll Cardiol. (2019) 73:2647–55. 10.1016/j.jacc.2019.03.483 31146808

[6] Edelman JJ Thourani VH. Commentary: transcatheter mitral valve-in-valve: not yet a replacement for surgery. J Thorac Cardiovasc Surg. (2022) 163:1813–4. 10.1016/j.jtcvs.2020.09.005 32977965

[7] Kilic A Acker MA Gleason TG Sultan I Vemulapalli S Thibault D. Clinical outcomes of mitral valve reoperations in the United States: an analysis of the society of thoracic surgeons national database. Ann Thorac Surg. (2019) 107:754–9. 10.1016/j.athoracsur.2018.08.083 30365952

[8] Writing Committee M Otto CM Nishimura RA Bonow RO Carabello BA Erwin JP 3rd. 2020 ACC/AHA guideline for the management of patients with valvular heart disease: a report of the american college of cardiology/American heart association joint committee on clinical practice guidelines. J Am Coll Cardiol. (2021) 77:e25–197. 10.1016/j.jacc.2020.11.018 33342586

[9] Baumgartner H Falk V Bax JJ De Bonis M Hamm C Holm PJ. 2017 ESC/EACTS Guidelines for the management of valvular heart disease. Eur Heart J. (2017) 38:2739–91. 10.1093/eurheartj/ehx391 28886619

[10] Kamioka N Babaliaros V Morse MA Frisoli T Lerakis S Iturbe JM. Comparison of clinical and echocardiographic outcomes after surgical redo mitral valve replacement and transcatheter mitral valve-in-valve therapy. JACC Cardiovasc Interv. (2018) 11:1131–8. 10.1016/j.jcin.2018.03.011 29929633

[11] Belhaj Soulami R Castro M Haigron P Verhoye JP. Computer-assisted valve in valve in a deteriorated Mosaic valve using a library of bioprostheses. Catheter Cardiovasc Interv. (2020) 97:E893–E896. 10.1002/ccd.29395 33211370

[12] Eleid MF Whisenant BK Cabalka AK Williams MR Nejjari M Attias D. Early outcomes of percutaneous transvenous transseptal transcatheter valve implantation in failed bioprosthetic mitral valves, ring annuloplasty, and severe mitral annular calcification. JACC Cardiovasc Interv. (2017) 10:1932–42. 10.1016/j.jcin.2017.08.014 28982556

[13] Whisenant B Kapadia SR Eleid MF Kodali SK McCabe JM Krishnaswamy A. One-year outcomes of mitral valve-in-valve using the SAPIEN 3 transcatheter heart valve. JAMA Cardiol. (2020) 5:1245–52. 10.1001/jamacardio.2020.2974 32745164 PMC7391176

[14] Bouleti C Fassa AA Himbert D Brochet E Ducrocq G Nejjari M. Transfemoral implantation of transcatheter heart valves after deterioration of mitral bioprosthesis or previous ring annuloplasty. JACC Cardiovasc Interv. (2015) 8:83–91. 10.1016/j.jcin.2014.07.026 25616821

[15] Cheung A Webb JG Barbanti M Freeman M Binder RK Thompson C. 5-year experience with transcatheter transapical mitral valve-in-valve implantation for bioprosthetic valve dysfunction. J Am Coll Cardiol. (2013) 61:1759–66. 10.1016/j.jacc.2013.01.058 23500301

[16] Cheung AW Gurvitch R Ye J Wood D Lichtenstein SV Thompson C. Transcatheter transapical mitral valve-in-valve implantations for a failed bioprosthesis: a case series. J Thorac Cardiovasc Surg. (2011) 141:711–5. 10.1016/j.jtcvs.2010.11.026 21269643

[17] Yoon SH Whisenant BK Bleiziffer S Delgado V Dhoble A Schofer N. Outcomes of transcatheter mitral valve replacement for degenerated bioprostheses, failed annuloplasty rings, and mitral annular calcification. Eur Heart J. (2019) 40:441–51. 10.1093/eurheartj/ehy590 30357365

[18] Lu Y Yang Y Wang W Chen J Yin M Huang L. Transcatheter mitral valve-in-valve implantation with a new transcatheter heart valve for bioprosthetic degeneration. Front Cardiovasc Med. (2021) 8:783507. 10.3389/fcvm.2021.783507 35127858 PMC8811914

[19] Wei P Ma J Tan T Xie N Chen Z Zhang Y. A novel alternative: transapical transcatheter mitral valve-in-valve implantation using J-Valve for failed bioprosthesis. J Thorac Dis. (2021) 13:5055–63. 10.21037/jtd-21-975 34527343 PMC8411171

[20] Nishimura RA Otto CM Bonow RO Carabello BA Erwin JP 3rd Guyton RA. 2014 AHA/ACC guideline for the management of patients with valvular heart disease: a report of the american college of cardiology/American heart association task force on practice guidelines. Circulation. (2014) 129:e521–643. 10.1161/CIR.0000000000000031 24589853

[21] Liu X Tang Y Luo F Tian Y Li K Sun J. Transapical implantation of a self-expandable aortic valve prosthesis utilizing a novel designed positioning element. Catheter Cardiovasc Interv. (2017) 89:E30–7. 10.1002/ccd.26429 26811261

[22] Stone GW Adams DH Abraham WT Kappetein AP Genereux P Vranckx P. Clinical trial design principles and endpoint definitions for transcatheter mitral valve repair and replacement: part 2: endpoint definitions: a consensus document from the mitral valve academic research consortium. J Am Coll Cardiol. (2015) 66:308–21. 10.1016/j.jacc.2015.05.049 26184623

[23] Suri RM Schaff HV Dearani JA Sundt TM. 3rd Daly RC Mullany CJ. Determinants of early decline in ejection fraction after surgical correction of mitral regurgitation. J Thorac Cardiovasc Surg. (2008) 136:442–7. 10.1016/j.jtcvs.2007.10.067 18692655

[24] Pandis D Sengupta PP Castillo JG Caracciolo G Fischer GW Narula J. Assessment of longitudinal myocardial mechanics in patients with degenerative mitral valve regurgitation predicts postoperative worsening of left ventricular systolic function. J Am Soc Echocardiogr. (2014) 27:627–38. 10.1016/j.echo.2014.02.008 24735653

[25] Enriquez-Sarano M Tajik AJ Schaff HV Orszulak TA McGoon MD Bailey KR. Echocardiographic prediction of left ventricular function after correction of mitral regurgitation: results and clinical implications. J Am Coll Cardiol. (1994) 24:1536–43. 10.1016/0735-1097(94)90151-1 7930287

[26] Medvedofsky D Aronson D Gomberg-Maitland M Thomeas V Rich S Spencer K. Tricuspid regurgitation progression and regression in pulmonary arterial hypertension: implications for right ventricular and tricuspid valve apparatus geometry and patients outcome. Eur Heart J Cardiovasc Imaging. (2017) 18:86–94. 10.1093/ehjci/jew010 26873457

[27] Sadeghi HM Kimura BJ Raisinghani A Blanchard DG Mahmud E Fedullo PF. Does lowering pulmonary arterial pressure eliminate severe functional tricuspid regurgitation? Insights from pulmonary thromboendarterectomy. J Am Coll Cardiol. (2004) 44:126–32. 10.1016/j.jacc.2003.12.058 15234420

